# Addressing Blood–Brain Barrier Impairment in Alzheimer’s Disease

**DOI:** 10.3390/biomedicines10040742

**Published:** 2022-03-22

**Authors:** Chanchal Sharma, Hanwoong Woo, Sang Ryong Kim

**Affiliations:** 1 School of Life Sciences, Kyungpook National University, Daegu 41566, Korea; chanchalmrt@gmail.com; 2 BK21 FOUR KNU Creative BioResearch Group, Kyungpook National University, Daegu 41566, Korea; 3 Brain Science and Engineering Institute, Kyungpook National University, Daegu 41404, Korea; hwwoo@knu.ac.kr

**Keywords:** blood–brain barrier, aging, peripheral inflammation, Alzheimer’s disease

## Abstract

The blood–brain barrier (BBB) plays a vital role in maintaining the specialized microenvironment of the brain tissue. It facilitates communication while separating the peripheral circulation system from the brain parenchyma. However, normal aging and neurodegenerative diseases can alter and damage the physiological properties of the BBB. In this review, we first briefly present the essential pathways maintaining and regulating BBB integrity, and further review the mechanisms of BBB breakdown associated with normal aging and peripheral inflammation-causing neurodegeneration and cognitive impairments. We also discuss how BBB disruption can cause or contribute to Alzheimer’s disease (AD), the most common form of dementia and a devastating neurological disorder. Next, we document overlaps between AD and vascular dementia (VaD) and briefly sum up the techniques for identifying biomarkers linked to BBB deterioration. Finally, we conclude that BBB breakdown could be used as a biomarker to help diagnose cognitive impairment associated with normal aging and neurodegenerative diseases such as AD.

## 1. Introduction

Normal brain function requires an adequate supply of blood as well as the anatomical and functional integrity of blood vessels, as they are necessary for transporting oxygen and nutrition, removing CO_2_ and other waste products, and thus maintaining body homeostasis [1]. The blood–brain barrier (BBB) comprises microvascular endothelial cells lining the cerebral capillaries that penetrate the brain and spinal cord, thus forming a well-developed central nervous system (CNS) [2]. Moreover it, separates the blood from the brain parenchyma and regulates the delivery of energy metabolites and nutrients to the neurons, essential for synaptic functioning [3]. Along with the cerebrospinal fluid (CSF), the BBB also prevents the free paracellular diffusion of water-soluble molecules via an elaborate network of complex tight junctions that interconnect the endothelial cells [4]. Furthermore, a functional BBB is characterized by several permanently active transport mechanisms, which are specifically expressed by brain capillary endothelial cells to ensure the transport of nutrients into the CNS while excluding blood-borne molecules that could be detrimental to the milieu required for neural transmission [4,5,6]. Meanwhile, if the BBB is disrupted, blood-derived neurotoxic proteins, such as fibrin, thrombin, hemoglobin, iron-containing hemosiderin, free iron, and/or plasmin (an extracellular matrix [ECM]-degrading enzyme), accumulate in the CNS. This results in progressive neurodegeneration and neuron loss mediated by direct neuronal toxicity, oxidative stress, and/or detachment of neurons from their supporting ECM [2,7].

**Figure 1:**
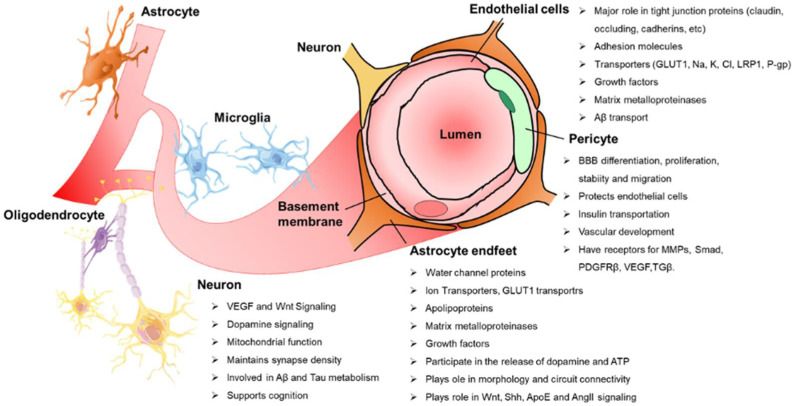
**The cellular and molecular components required for BBB formation, maintenance, and function.** Pericytes, endothelial cells, astrocytes, neurons, and microglia make up the neurovascular unit. Pericytes share a common basement membrane with the endothelium and connect with several transmembrane junction proteins. Low-level bulk-flow transcytosis and tight junction and adherens junction proteins between endothelial cells maintain the BBB integrity. Astrocytes connect with pericytes, endothelial cells, and neurons. Microglia regulate immune responses.

Several cell types collaborate through continuous crosstalk to maintain the BBB and regulate cerebral blood flow (CBF) (Figure 1). Together, endothelial cells forming the inner layer of vessel walls, mural cells lining the vessels assisting and regulating the vascular tone (pericytes and vascular smooth muscle cells [SMCs]), and astrocytes with end-feet covering much of the vasculature make up the neurovascular unit (NVU) [8]. The NVU also contains other glial cells, such as oligodendroglia and microglia, neurons, and peripheral immune cells, which all participate in this biological interaction [2]. At the molecular level, the integrity of the cerebrovascular system is aided by various gap junction proteins, such as claudins, occludin, zonula occludens (ZO), and connexins, and cell adhesion molecules, such as vascular endothelial cadherin and platelet endothelial cell adhesion molecule between endothelial cells, pericytes, and astrocytes [8]. These gap junction proteins and cell adhesion molecules restrict the paracellular and transcellular diffusion of molecules into the CNS [5,9,10]. This property of low paracellular permeability is primarily controlled by tight junction proteins, which prevent paracellular transmission between apposing brain microvascular endothelial cells [11]. The BBB is located at the center of the NVU and consists of a monolayer of firmly sealed endothelial cells running along the vascular tree with low paracellular and transcellular permeability [9]. Under normal conditions, this mainly precludes the extravasation of any solutes (big or small) (unless particular transporters are present) as well as the migration of any type of blood-borne cell. However, BBB rupture can increase paracellular permeability, which allows leukocytes to enter the brain tissue and contributes to edema. In parallel, alterations in the endothelium pinocytotic vesicular system can result in the uptake of fluid and macromolecules and their transfer into the brain parenchyma [9]. Thus, upholding the endothelial barrier is essential for the specialized transport properties and functions of the BBB, as it also prevents potentially neurotoxic plasma components, blood cells, and infections from entering the brain [7]. Moreover, these cells express multiple transport systems required to transport nutrients, energy metabolites, and other essential molecules from the blood to the brain and transport metabolic waste products from the brain’s interstitial fluid (ISF) into the blood [7,12]. Thus, the BBB functions as a crucial nervous system homeostatic site, connecting the CNS, systemic circulation, and major body systems—such as the respiratory, renal, hepatic, and immunological systems.

As the BBB is vital to maintaining the microenvironment of the CNS, any impairment in the cellular or molecular components of the BBB can cause neurodegenerative diseases [3]. BBB disruption reduces oxygen and glucose delivery, potentially resulting in hypoxia and hypoxia-induced inflammation [13,14]. The ensuing pathological processes make the brain more vulnerable to neuronal malfunction and neurodegeneration. Thus, effective neuronal functioning and healthy aging require the management of the transport of numerous chemicals between the circulation and the brain, as well as neurotransmitter recycling [15]. The BBB assists CSF and aids in waste clearance by restricting the passage of various chemicals from the brain parenchyma while providing substrates for brain cell metabolism [15,16,17]. Both the BBB and blood-CSF barriers assist in the regulation of ionic composition (that of Na^+^, Cl^−^, K^+^, Ca^2+^, and Mg^2+^) and the volume of CSF and ISF, which is necessary for brain cells to function properly [16]. The BBB helps to preserve the ionic composition of the ISF by separating the blood in the vasculature from the ISF and cells within the brain parenchyma [15,17]. CSF fills the larger gaps within and around the CNS, while ISF surrounds the parenchymal cells of the brain and spinal cord. Any defect in the CNF outflow can limit the clearance of ISF solutes from the CNS parenchyma [15]. This may result in the entry of peripheral immune cells in the CNS parenchyma. During pathophysiological situations, they can cross the BBB, from the perivascular area through the glia limitans. This, in turn, limits the removal of ISF from the brain, potentially leading to the accumulation of toxic waste products such as amyloid-β (Aβ) in Alzheimer’s disease (AD) and α-synuclein in Parkinson’s disease (PD) [18,19]. 

However, the stage at which BBB breakdown occurs in the living human brain and whether it contributes to cognitive impairment remains elusive. In this review, we first briefly discuss the pathways that play an essential role in maintaining and regulating BBB integrity. Second, we discuss the causes, characteristics, and consequences of BBB breakdown by emphasizing the aging process, and then review the characteristic features and mechanisms of BBB breakdown associated with normal aging that further cause neurodegeneration and cognitive impairments. Next, we summarize and discuss the phenotypes and mechanisms of BBB disruption correlated with cognition decline in AD. Subsequently, we discuss the overlap between AD and vascular dementia (VaD). We also sum up biomarkers associated with BBB breakdown and various techniques to identify them. Finally, we conclude that BBB breakdown could be a novel biomarker to diagnose cognitive impairment associated with normal aging and neurodegenerative disorders such as AD.

## 2. Cellular and Noncellular Architecture of the BBB

The BBB was accidentally discovered in the late 19th century by a German physician named Paul Ehrlich, who found that tracer dye injected into mouse circulation infiltrated all tissues except the brain and spinal cord [20]. The meninges, which are three concentric connective tissue membrane layers, serve as a functional barrier surrounding the brain and spinal cord to protect and support the CNS [21]. The layers include pachymeninx, also called the dura mater and the leptomeninges that comprise the arachnoid and pia mater [21,22]. These layers help to safeguard the CNS by covering it with a protective layer and to establish the BBB [21]. 

The dura mater, which is formed of dense fibrous tissue and is approximately 1-mm thick in humans, forms the outermost layer of the meninges [23]. The outer layer projects vascular connections into the bone and is linked to the inner surface of the skull. It contains arteries, veins, and lymphatics that are distinct from the CNS. Dural capillaries are fenestrated and do not have tight junctions. Thus, they do not participate in establishing the BBB. The arachnoid mater is a 200-mm thick middle layer, which is impermeable to fluids and abuts the inner aspect of the dura, producing a border layer of cells linked by tight junctions and expressing efflux pumps [24]. The arachnoid mater acts as a real blood–CSF barrier by forming a barrier between the dura mater and the CSF-drained subarachnoid space (SAS). A one-cell-thick delicate layer of pia mater which is permeable to solutes and immune cells but not to erythrocytes protects the CNS surface [25]. The pia mater, which is reflected from the surface of the CNS and divides the SAS from the CNS and perivascular compartments, covers arteries and veins in the SAS [26]. Additionally, the surface of the CNS is coated by the glia limitans, comprising compacted astrocytic end-feet and an overlaying parenchymal basement membrane. Unlike the dura mater, the pia mater does not have tight junctions [27]. The CSF allows fluid and low-molecular-weight tracers to move readily through the glia limitans, but T-cell trafficking is restricted [28,29].

The BBB is made up of two key components that act as a barrier between the blood and the CNS parenchyma. A BBB for solutes is generated by complex tight junctions connecting specialized capillary endothelial cells with limited pinocytotic activity [5]. A BBB, controlling the immune cell access into the CNS, is found in postcapillary venules [30]. T cells must first pass the vascular endothelium and then the glia limitans to reach the CNS parenchyma from the blood [30]. The fully differentiated BBB is a complex system that comprises highly specialized endothelial cells and their underlying basement membrane (enclosing a large number of pericytes), and perivascular antigen-presenting cells, surrounded by a parenchymal basement membrane [4]. The morphological correlate of the endothelial barrier constitutes continuous belts of tight junctions between adjacent overlapping endothelial cells [20]. The intracellular gap between the overlapping endothelial cells is sealed to sufficiently prevent paracellular leakage under normal physiological conditions.

### 2.1. Pathways Regulating BBB Integrity

The complex cellular and non-cellular extracellular matrix components of the BBB work in concert to maintain BBB integrity, and impairments in any one of these can cause BBB malfunction and disintegration. For instance, various tight junctions and adherens junctions are vital to BBB formation and maintenance [2]. Endothelial cells prevent thrombosis and preserve permeability, homeostasis, and vascular wall integrity [7]. Meanwhile, pericytes help maintain the integrity and proper permeability of the BBB by preserving the tight junctions of endothelial cells (e.g., claudin-5, occludin, and ZO-1) and regulating transcytosis in endothelial cells [31,32,33]. Astrocytes secrete crucial BBB maintenance factors such as sonic hedgehog (Shh), vascular endothelial growth factor (VEGF), angiopoietins-1 (Ang-1), angiotensin-converting enzyme-1 (ACE-1), glial-derived neurotrophic factor, and apolipoprotein (ApoE) [34]. A neural VEGF deficiency causes aberrant vascular density and other abnormalities, mainly in the cortex and retina [35]. The VEGF receptor regulates the Ras–Raf–MEK pathway (which promotes endothelial cell proliferation), the PI3K–AKT system (which promotes endothelial cell survival), and the p38 MAPK–HSP27 route (which promotes endothelial cell migration) [36]. Additionally, in CNS endothelial cells, the Wnt–β-catenin pathway regulates several genes, such as Lef1, Apcdd1, Axin2, Stra6, and Slc2a1 through β-catenin [37]. Wnt induces the expression of BBB genes, including those encoding nutrient transporters (such as Slc2a1, encoding Glut-1), which induces BBB functions [38]. Thus, an absence of the signaling elements downstream of β-catenin in CNS endothelial cells results in the failure of vessel formation and substantially disrupts the BBB [37]. According to a recent study, increased β-catenin levels in the developing brain induce the expression of the death receptors Dr6 (also known as tumor necrosis factor receptor superfamily member 21, Tnfrsf21) and Troy (also known as Tnfrsf19), known to interact with downstream elements of the VEGF pathway and interferes with endothelial cells sprouting and BBB formation [39]. Besides, β-catenin regulates tight junction formation [38]. Furthermore, the Shh pathway, which provides a barrier-promoting effect and an endogenous anti-inflammatory balance to CNS-directed immune attacks, has been recognized as a key player in the development of BBBs [40]. Phenotypic abnormalities in Shh-knockout mice are associated with BBB formation abnormalities linked with lower occludin and claudin-5 expression levels [40]. Moreover, a low expression of these tight junction proteins was also associated with plasma protein leakage that impaired the BBB [40]. 

Inflammation activates microglia, the immune cells of the central nervous system, and polarizes them into the M1 (cytotoxic) or M2 (neuroprotective) phenotype; thus, microglia can harm or protect the BBB [41,42]. When activated by injury or insult to the brain, microglia proliferate, undergo morphological changes, and secrete chemokines and cytokines. Besides, ECM influences cell–cell and cell–matrix interactions inside the NVU, regulating the BBB’s shape and function [43].

### 2.2. BBB vs. Blood–CSF Barrier Alteration

The CNS is tightly sealed from the changeable milieu of the blood by the BBB and blood–CSF barrier. Both barriers are regarded as essential components of neurovascular coupling as well as active surveillance and coordinated immune responses [44,45]. Although in principle both barriers serve the same defensive purpose for the CNS, their distinct structures allows the exchange of various substances between the bloodstream and brain. The blood–CSF barrier is found in the choroid plexus of each ventricle of the brain, inhibiting the paracellular diffusion of water-soluble molecules [4]. The choroid plexus is a spongy, vascularized tissue linked to the ventricle walls that produces CSF. Unlike the BBB, the blood–CSF barrier is also present at the epithelial level, wherein capillaries are relatively leaky and permeable to small molecules, thereby allowing, among other processes, the rapid delivery of water through the bloodstream to the surrounding epithelial cells for CSF production in the choroid plexus [46]. These multiple transport mechanisms allow the guided transit of ions and nutrients into the CSF as well as the removal of toxic substances from the CSF, thereby maintaining the barrier and secretory function of the epithelial cells in the choroid plexus.

The blood–CSF barrier is reportedly altered in CNS pathologies, resulting in the development of edema and the migration of inflammatory cells into the CNS [27]. At steady-state, peripheral immune cells do not reach the CNS parenchyma. However, during pathophysiological situations (such as autoimmune illness, injury, stroke, or infection), they can cross the BBB, but only after acquiring the ability to travel from the perivascular area through the glia limitans. 

## 3. Causes, Characteristics, and Consequences of BBB Breakdown

Tight junction proteins, endothelial cells, cellular transport pathways, and enzymatic machinery all work together and contribute to normal BBB maintenance, function, and integrity. However, aging, peripheral inflammation, systemic diseases, and ischemic injury can disrupt these processes, disrupting BBB function and integrity. This results in ionic imbalance, plasma proteins leaks, and microglia and astrocytes activation, producing various cytokines and chemokines. These damage mechanisms work in concert, reinforcing each other to drive the pathology in the aging brain, contributing to BBB breakdown and disruption by triggering or exacerbating other pathogenic processes. They ultimately accelerate neuronal degeneration and cognitive decline through neuronal malfunction.

### 3.1. Aging: The Major Culprit for BBB Disruption

Normal aging in the brain and BBB reduces age-associated physical activity without necessarily impairing cognition, but it increases the frequency of age-related disorders such as AD and PD [47]. Even in the absence of neurological diseases, BBB disintegration is a typical aspect of the aging process. Moreover, age-related BBB degradation makes the brain more susceptible to neuronal impairment and cognitive decline [48]. A study on healthy older adults who underwent dynamic contrast-enhanced magnetic resonance imaging (DCE-MRI) with an intravenously administered gadolinium-based contrast agent revealed substantial BBB leakage concentrated in the brain regions most prone to aging damage [47]. Recent studies have also demonstrated the potential of BBB breakdown as a biomarker for the aging process and to identify age-related disorders [49]. Similarly, Montagne and colleagues documented an early BBB disintegration occurrence in the aging human brain starting in the CA1 and dentate gyrus regions of the hippocampus that may contribute to cognitive impairment [50].

The BBB breaks down as the brain ages; as a result, its permeability increases, and CBF decreases. Furthermore, the neovascularization potential and the capillary density in the brain vasculature decrease [51]. Besides, changes in ECM, which maintains BBB integrity by inducing occludin (a tight junction protein) expression, increase BBB permeability. Thus, blood-derived toxic and inflammatory substances infiltrate the brain and change the neuronal biochemical microenvironment, leading to neurodegenerative diseases [8,52,53,54]. Pericyte loss has also been observed in elderly mouse, rat, and human brains [55,56]. Furthermore, pericyte-mediated BBB disruption has been linked to high soluble platelet-derived growth factor receptor beta (PDGFRβ) levels in the CSF [57]. The end-feet of astrocytes sheathing pericytes also plays a role in BBB formation and maintenance during aging. With age, astrocyte end-feet vascular coverage and aquaporin-4 (AQP4) expression decrease, and glial fibrillary acidic protein expression increases, thus increasing astrogliosis [58,59]. Similarly, activated microglia produce tumor necrosis factor-alpha (TNF-α), proteases, nitric oxide (NO), and peroxide, which affect tight junction proteins throughout aging [60]. This change causes BBB leakage, which results in cell damage and neurodegeneration [60,61]. Besides, BBB breakdown and impairment are also caused by a decrease in the interaction of neurons with endothelial cells and astrocytes, which increase the BBB’s permeability to albumin [62].

Blood-derived proteins such as fibrinogen and plasminogen can also cross the compromised BBB and the pro-inflammatory fibrin aggregates in the brain, activating microglia, increasing reactive oxygen species (ROS) production, and activating the nicotinamide adenine dinucleotide phosphate (NADPH) oxidase. Next, NADPH oxidase upregulates the pro-inflammatory gene expression, damaging neuronal axons and triggering a decline in cognition [63,64]. In addition, complement proteins cross the BBB, which reportedly alters the function of microglia, oligodendrocytes, and neurons, allows inflammatory cells to infiltrate the brain, and induces cytokine cascades contributing to neurodegeneration [65,66,67]. Likewise, during aging, oxidative stress upregulates the expression of various cytokines and chemokines, such as matrix metalloproteinase 3 (MMP3) and p16INK4A (senescence-associated secretory phenotype [SASP]) in astrocytes, inducing BBB disruption, neuroinflammation, and cognitive impairments [68,69,70]. Besides, it activates microglia, which release cytokines, chemokines interleukin (IL)-6, IL-1β, and TNF-α, elevating ROS and the production of reactive nitrogen species (RNS), ultimately disrupting the BBB [71]. In addition, age reduces the capability of neurons to clear ROS and RNS, resulting in BBB degradation and neurodegeneration [71]. Although BBB integrity disruptions appear multi-factorial, age-related impairments in cellular transport suggest that several functional pathways contribute to BBB deterioration. Nonetheless, animal research provides a path for investigating the relationship between aging, neurovascular disease, and neurodegeneration in humans.

### 3.2. Peripheral Inflammation

Peripheral inflammation is essentially an organism’s defensive response. However, excessive and dysregulated inflammation has negative consequences. For example, cytokines, including IL-1β, IL-6, IL-9, IL-17, interferon-γ, TNF-α, and CCL2, can reduce the expression of claudin-5, the most important tight junction protein, responsible for the selective permeability of the BBB. In addition, the degradation of occludin, ZO-1, and ZO-2, other tight junction proteins, was also associated with lipopolysaccharide (LPS)-induced systemic inflammation [72,73,74,75]. Since tight junctions are one of the most critical BBB components, any change in tight junction proteins expression directly impacts the BBB. Henceforth, nowadays, tight junction variations are frequently utilized as indications of BBB failure. Furthermore, LPS has a direct cytotoxic effect on the BBB endothelium by blocking the P-glycoprotein function and increasing the release of matrix metalloproteins (MMPs), resulting in membrane anomalies, endoplasmic reticulum stress, mitochondrial damage, and cell death [76]. This leads to endothelial cell disintegration, and BBB damage facilitates the entry of neurotoxic chemicals into the CNS, raising the risk of neurodegenerative disease [76,77]. The upregulation of the cell adhesion molecules on endothelial cells, such as vascular cell adhesion molecule 1 (VCAM-1), intercellular adhesion molecule 1 (ICAM-1), and E-selectin, is another effect of peripheral inflammation which allows peripheral immune cells to enter the CNS, as in aging and chronic inflammation [75]. Furthermore, IL-1β can increase the α5 integrin-dependent adhesion of endothelial cells, affecting the BBB integrity by changing the cell–cell junctions and cell–matrix adhesion [73]. In such inflammatory situations, astrocytes release VEGF-A, increasing endothelial nitric oxide synthase signaling, reducing occludin and claudin-5 expression, and facilitating the entry of peripheral lymphocytes into the CNS [78,79].

M1 microglia perform pro-inflammatory signaling through the toll-like receptor (TLR)-4, the interferon-γ receptor complex, the granulocyte-macrophage colony-stimulating factor (GM-CSF) receptor, and COX2 [80,81,82]. Besides, there is ROS production and oxidative stress involved in M1 microglia, which elevates in response to the increased expression of iNOS during peripheral inflammation, contributing to BBB disruption [83]. Peripheral inflammation also affects many transport routes: it downregulates efflux transporters of Aβ, anions, and amino acids and upregulates influx transporters of insulin, monoamines, and lysosomal enzymes [84]. Furthermore, the cerebral endothelium expresses circulating cytokines, such as IL-1, IL-6, and TNF-α, which can directly activate the endothelium and disrupt the BBB [85].

## 4. AD

### 4.1. Clinical and Neuropathological Characteristics

AD is characterized by a selective neuronal vulnerability resulting in a progressive loss of memory [86]. The symptoms of AD, such as moderate forgetfulness that affects short-term memory, resemble those of normal aging when they first arise. However, long-term memory is also impacted as the disease progresses, resulting in difficulties recalling events, activities, and names of familiar people or things, and performing daily tasks. The onset of symptoms (usually over 8–10 years) corresponds to the progression of degenerative changes in the brain, which include—(1) the formation of dystrophic neurites around a central core of amyloid beta (Aβ plaques); (2) the formation of abnormal filaments made up of a highly phosphorylated form of the microtubule-associated protein tau (neurofibrillary tangles; NFTs) in the perikaryon of certain neurons, as well as neuropil threads in axons and nerve terminals; and (3) loss of vulnerable neurons, primarily pyramidal, cholinergic, noradrenergic, and serotonergic neurons [86,87]. As the understanding of the pathological changes that occur in AD improved, research has shifted to the investigation of more specific modifications in Aβ processing, such as the cleavage of amyloid precursor protein (APP) into Aβ peptides (Aβ1–40 and Aβ1–42) and the relevance of Aβ oligomers (small aggregates of 2–12 peptides) [86,88]. The Aβ1–42 peptide aggregates more readily than the Aβ1–40 peptide, and the ratio of the two isoforms is regulated by the pattern of APP by three different secretases, namely; α, β, and γ secretases [88]. Aβ56 appears to be a peptide of particular interest because it was negatively related to cognitive decline in an APP mouse model and induced memory problems when injected into the rat brain [89]. 

The main component of NFTs is tau, a microtubule-associated protein. Although tau is a soluble protein, it forms insoluble aggregates during the formation of NFTs, which damage the neuronal structure and function. Tau monomers create oligomers, which organize into a β sheet before forming NFTs. Although the pathways linking Aβ and tau are not clearly understood, the amyloid cascade indicates that changes in tau and the consequent formation of NFTs are triggered by toxic concentrations of Aβ [90]. Once the filamentous tau has formed, it can spread to other parts of the brain. Apart from these classical pathological hallmarks of AD, numerous other potentially modifiable factors can contribute to the clinical manifestation of AD. For instance, aging hampers Aβ clearance or may result in the overproduction of Aβ. This, in turn, results in the deposition of Aβ in blood vessel membranes [91]. In general, excessive Aβ is extruded by BBB transporter systems such as the P-glycoprotein transporter (P-gp) and low-density lipoprotein (LDL) receptor. However, if transporters are disrupted as a result of Aβ accumulation, then microglia activation may be induced, and the release of ROS, complement proteins, and proinflammatory cytokines may be triggered [92,93]. Altogether, this induces neurotoxicity and tau hyperphosphorylation [92]. The hyperphosphorylated tau forms aggregates leading to the formation of NFTs [94]. 

The LDL receptor family has a strong affinity for human apolipoprotein E (APOE) and its member exhibit APOE isoform (APOE2, APOE3, and APOE4)-specific binding affinity [95]. Compared with the typical ApoE3 allele, polymorphism in the APOE gene is a major genetic risk predictor for late-onset AD, with the ApoE4 allele providing an elevated risk and the ApoE2 allele conferring a decreased risk [96]. Many Aβ-dependent and Aβ-independent pathways are differently regulated by ApoE isoforms. Accumulating evidence suggests that ApoE and LDL receptors have a functional connection that influences the risk of developing VaD and AD. Hippocampal ApoE levels positively correlate with long-term spatial memory retention in mice with human LDL receptors [95]. Moreover, ApoE apparently alters tau pathology, tau-mediated neurodegeneration, and microglial responses to AD onset. ApoE4 is either harmful or inefficient in many brain homeostatic mechanisms, including lipid transport, synaptic integrity and plasticity, glucose metabolism, and cerebrovascular function [96]. 

### 4.2. Transgenic Mouse Models of AD

Genetically modified animal models have contributed to the discovery of pathogenic pathways involved in AD and the preclinical testing of prospective treatment approaches. However, AD is currently incurable, it is important to establish and characterize appropriate animal models to aid translational research and preclinical drug development. Table 1 summarizes some of the most extensively utilized transgenic mouse strains in AD research, which are discussed in the present review.

## 5. The Implication of BBB Dysfunction in AD

**Figure 2:**
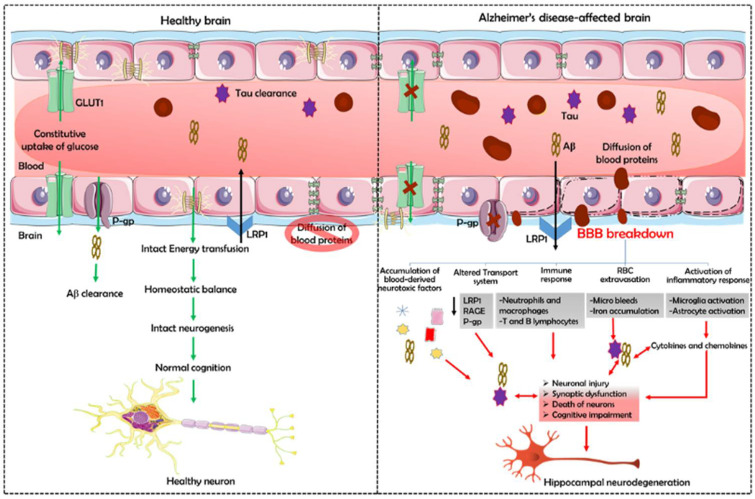
**The blood–brain barrier in healthy and Alzheimer’s disease-affected brains.** Tight junction protein disruption, decreased transport function, increased permeability, pericyte degradation, and accumulation of toxic substances such as Aβ and tau due to impaired clearance disrupt the BBB. These events cause an accumulation of neurotoxic hemoglobin and iron in the brain, increasing ROS production and oxidative stress in neurons. Neuronal toxic blood-derived proteins, such as fibrinogen, thrombin, and plasminogen, degrade the neuronal extracellular matrix, resulting in cell death and detaching neurons. This activates microglia and promotes neuroinflammation. BBB failure can also abolish the immune privilege, leading to the production of anti-brain antibodies directed against various axonal and membrane components of neurons.

The BBB prevents blood-derived products, pathogens, and cells from penetrating the brain, which is essential for normal neuronal functioning and information processing (Figure 2). Both post-mortem and brain imaging studies have shown BBB damage and accumulation of blood-derived proteins in the hippocampus, cortex, and cerebrospinal fluid [100,101]. Besides, experiments in murine models have shown that BBB breakdown leads to tissue accumulation of potentially neurotoxic blood-derived products (e.g., immunoglobulins, albumin, fibrinogen, thrombin, and prothrombin kringle-2) that may or may not normally enter the brain but can damage neurons with increased BBB permeability within the hippocampus [100,102,103].

### 5.1. Neurovascular Unit Dysfunction

The endothelium serves as one of the essential components of the NVU. And endothelium degeneration, resulting in BBB dysfunction, is one of the many causes of AD. Reports suggest a crucial role of MMP-9 in the disruption of TJ proteins, ECM degradation, activation of the immune system, and the development of neuroinflammation resulting in endothelial barrier failure [104,105]. Moreover, all these processes were associated with the insoluble Aβ levels in post-mortem human cortices [106]. Furthermore, the transcriptome profiling of endothelial cells revealed a low mesenchyme homeobox 2 (MEOX2, required for vascular differentiation) expression in AD-affected brains [107]. The authors of this report showed that restoring MEOX2 protein expression in endothelial cells from AD patients stimulated angiogenesis, transcriptionally suppressed AFX1 forkhead transcription factor-mediated apoptosis, and increased the levels of low-density lipoprotein receptor 1 (LRP-1), a major Aβ clearance receptor, at the BBB [107]. Another transcriptome study demonstrated that a pro-inflammatory response governs the generation of angiogenic endothelial cells and that endothelial cells may play a role in the altered angiogenesis and immune response in AD development [108]. 

In addition, astrogliosis and astrocyte degradation also occur in AD patients’ brains. According to one study, A1 astrocytes are prevalent during the early stages of cerebral amyloid angiopathy in mice with AD, and they secrete neurotoxins that cause cell death and inflammation [109]. Further, the alteration of AQP-4 may result in vascular dysfunction in AD patients [58]. Moreover, AD patients have a reduced pericyte coverage on brain capillaries, which is negatively correlated with the BBB permeability [110]. Besides, AD patients carrying ApoE4 alleles had a more severe reduction than those with ApoE3 alleles, suggesting that ApoE variants differently affect pericyte attachment to endothelial cells [111]. Moreover, in an in vitro BBB model, ApoE3 pericytes had a more potent endothelial barrier integrity reduction effect than ApoE4 pericytes [112]. These findings suggest that ApoE isoforms can alter BBB integrity by modulating the endothelial gene expression in various ways. Besides, pericytes with the ApoE4 variation lose their ability to support and regulate endothelial cells, contributing to vascular dysfunction.

### 5.2. Accumulation of Neurotoxic Blood-Derived Products

Tibbling and coworkers established the immunoglobulin G (IgG) index, used to assess antibody synthesis and as an index of immune function/inflammation within the CNS, and the CSF/serum albumin ratio, which indicates BBB rupture [113,114]. The CSF/serum ratios were elevated in both patients with AD and patients with multi-infarct dementia as compared to their age-matched controls [115]. Furthermore, ApoE3 negative patients also had high CSF/serum ratios [116]. Besides albumin and IgG, elevated levels of peptides related to or produced from hemoglobin were found in the cerebellum of AD patients [117]. Further, fibrin was also found to be accumulated in amyloid-positive arteries in AD brains, and fibrin removal played a role in protecting mice from neuroinflammation and cognitive deficits [118,119].

Normal brains do not produce prothrombin, but AD patients’ brains contain high prothrombin levels, indicating a leakage across a disrupted BBB [120]. This plasma glycoprotein and zymogen of thrombin is cleaved by factor Xa into prothrombin kringle-1 and 2 and active thrombin induces blood coagulation [121]. Increased active thrombin levels have been linked to hippocampus neuron degeneration [122]. The authors of this study suggested that microglial NADPH oxidase-mediated oxidative stress causes thrombin-induced neurotoxicity in the hippocampus in vivo. We recently assessed the role of prothrombin kringle-2 and found elevated levels in the hippocampi of AD patients and 5XFAD mice [102]. Here, we identified a novel pathological mechanism of AD mediated by an abnormal accumulation of prothrombin kringle-2, which functions as an important pathogenic factor in the adult brain via BBB breakdown. Treating the 5XFAD mice with rivaroxaban or adding caffeine to their water supply prevented prothrombin kringle-2 upregulation and preserved the BBB. Furthermore, preventing prothrombin kringle-2 upregulation in 5XFAD animals reduced neurotoxic symptoms such as hippocampus neurodegeneration and object recognition impairment caused by neurotoxic inflammatory responses [102].

Patients with preclinical and clinical AD symptoms also suffer from cerebral microbleeds and iron buildup [123,124,125]. Recently, Wu et al. explored how lead exposure through the BBB could aggravate AD progression in mice [126]. Lead exposure raises the blood lead concentration, accelerating Aβ1–42 deposition in APP/PS1 mouse cortices and affecting ZO-1 and claudin-5 protein levels. Besides, lead exposure increases phosphorylated tau (p-tau) expression in C57BL/6J and APP/PS1 mice and decreases the mRNA and protein expression levels of LRP-1, the major peripheral Aβ-sequestering agent [126]. Recently, He and coworkers discovered that microvascular endothelial cells with altered LRP-1 and receptor for advanced glycation end products (RAGE) expression dysregulate Aβ transport across the BBB [127]. RAGE is involved in Aβ transport in the brain and spreads its toxicity. Additionally, Aβ and p-tau pathology is also influenced by white matter lesions and microhemorrhages, dyslipidemia, altered brain insulin signaling, and insulin resistance, as well as oxidative stress, mitochondrial damage, inflammation, and hypoperfusion [127]. Besides, poor angiogenesis and inefficient vascular regeneration can degrade the brain endothelium in AD [128].

### 5.3. Accumulation of Aβ in Cerebrovasculature

Aβ is enzymatically destroyed in numerous cell types in the brain, including endothelial cells, pericytes, astrocytes, neurons, and microglia, by neprilysin, insulin-degrading enzyme, tissue plasminogen activator, and matrix metalloproteins MMPs) [129,130,131,132]. The BBB is also an underlying source of Aβ peptides, which downregulate ZO-1, occludin, and claudin-5 and increase the permeability of endothelial cells by interacting with RAGE, activating MMPs, and stimulating oxidative stress pathways [133,134]. LRP1 and/or another lipoprotein receptor are involved in the cellular clearance of the Aβ peptide by astrocytes and VSMCs [135]. In addition, microglia play a crucial role in amyloid-directed immunotherapy and lower the amyloid load in the brain by clearing amyloid aggregates [136]. Passive ISF–CSF bulk flow and subsequent CSF clearance could account for 10%–15% of total amyloid elimination [137].

Furthermore, 4-month-old Tg2576 mice had lower BBB integrity in specific parts of the cerebral cortex than their non-transgenic littermates [138]. These animals also had a higher age-related albumin uptake in their brains than non-transgenic mice [138]. This study also revealed that BBB disruption in young Tg2576 mice (aged 4 to 10 months) led to Aβ entry into the brain. In another study, researchers looked at the expression of enzymes involved in ceramide and sphingolipid metabolism, as well as the dysregulation of de novo ceramide formation and S1P metabolism in the brains of mice with hyperhomocysteinemia [139]. The hydrophobic nature of these chemicals allows them to cross the BBB, provoke neurotoxic responses, elevate pro-inflammatory cytokines, and cause neurodegeneration in progressive AD associated with metabolic failure.

Moreover, BBB breakdown in AD causes insoluble extracellular Aβ plaques accumulation along the blood vessels walls, resulting in microbleeds and hemorrhages in the brain [123,124,125]. It also causes inflammation in the NVU [8]. The low number and density of pericytes in the cortex and hippocampus of AD patients lead to the upregulation of Aβ and p-tau proteins, causing microglial activation, neuroinflammation, neuronal degeneration, and cognitive impairment [140,141]. The study also revealed that AD patients have markedly lower pericyte coverage of capillaries in the cortex and hippocampus than non-AD controls, and this was correlated with reduced BBB integrity [141]. Another study that assessed the association between soluble platelet-derived growth factor receptor (sPDGFR) levels in the CSF, CSF albumin, and the CSF/serum albumin ratio, the reduced CSF Aβ42 levels, and higher CSF total tau and p-tau levels in AD, also revealed a positive correlation between pericyte injury and BBB collapse, linked to the severity of AD [101]. Besides, pericyte loss caused by faulty PDGF-β signal transduction from endothelium to PDGFR in pericytes causes BBB breakdown, CBF reductions, and hypoxia, leading to age-dependent secondary neuronal and synaptic changes associated with neuronal and synaptic dysfunction and ultimately to AD [55]. In vitro exposure to fibrillary Aβ1–42 experiments revealed an increased apoptotic activity and decreased pericyte proliferation, decreasing the overall pericyte survival [142]. 

Pericyte detachment from the capillary endothelium is also linked to the leakage of a variety of toxic serum proteins, endothelial cell death, capillary regression, and a decrease in regional CBF [55]. Cyclosporine A improved BBB integrity in ApoE-transgenic animals with AD by blocking an inflammatory pathway in pericytes that caused tight junction breakdown and extravasation of neurotoxic serum proteins [143]. Similarly, Sagare and coworkers showed that pericyte loss elevates Aβ40 and Aβ42 levels and accelerates amyloid angiopathy and cerebral β-amyloidosis in mice overexpressing the Aβ-precursor protein (APP) by decreasing soluble Aβ40 and Aβ42 clearance from brain ISF before Aβ deposition [144]. Furthermore, the authors showed that a pericyte deficit causes tau pathology and early neuronal death, resulting in cognitive decline in APP knockout transgenic mice, but not in APP overexpressing transgenic mice. These findings imply that pericytes govern numerous steps of the AD-like neurodegenerative pathogenic cascade and could thus be a new therapeutic target for slowing the progression of AD [144]. 

Besides pericyte death, Aβ accumulation in the vasculature may alter astrocyte morphology, resulting in BBB dysfunction. A recent neuroimaging study on living mice with methoxy-XO4-labeled vascular amyloid revealed that vascular amyloid deposits separated astrocyte end-feet from the endothelial vessel wall [94]. The authors concluded that, while astrocytes can still emit vasoactive chemicals, vascular amyloid deposits stiffen blood arteries and decrease their dynamic range [145]. They also uncovered a novel mechanism CBF reduction in AD patients. 

### 5.4. Accumulation of Phosphorylated Tau in the Cerebrovasculature

Another significant pathologic marker of AD is tau, which is inappropriately phosphorylated in the AD brain and accumulates to produce neurofibrillary tangles (NFTs). Several studies have linked tau to BBB disruption. For example, AD patients and the Tg2576 mice model displayed oligomeric tau deposits [146]. Tau depositions in blood vessels caused morphological changes, such as increased atrophic string capillaries and abnormalities on the capillary surface [147]. Aged tau-overexpressing mice (P301L transgenic mice, an AD model) display abnormal, spiraling blood vessel morphology, reduced blood vessel diameters, and increased overall blood vessel density in the cortex [148]. Furthermore, these arteries had a disrupted blood flow, with periods of restricted flow usually not seen in normal capillaries. These changes accompanied cortical atrophy and increased the expression of angiogenesis-related genes such as VEGF-a, Serpine1, and Plau in CD31-positive endothelial cells [148]. Altogether, these findings suggest that tau pathogenic alterations in neurons can affect brain endothelial cells and thus the microvasculature’s integrity [148,149]. 

A recent cohort study found that the degree of tau expression was inversely correlated with CBF [150]. CBF reduction is common in AD patients, and it is linked to cognitive deterioration. Tau is also linked to reduced claudin-5 and occludin levels in AD patients’ brains [151]. According to this research, NFT alters blood vessel morphology and vascular function. However, uncovering the interaction mechanisms between tau/NFT and the different BBB cell types requires more research.

### 5.5. Alzheimer’s Disease and Vascular Dementia Co-Occur

Because vascular pathology not only facilitates but also potentiates vascular deficits, the co-occurrence of AD and VaD, often known as mixed dementia, is a possibility [152]. AD and VaD have several clinical characteristics in common, including cognitive decline and neuropsychiatric symptoms linked to behavioral changes. According to a clinical study by Boyle and colleagues, only 9% of 1000 individuals with cognitive deficits had pure AD pathology. Nevertheless, AD pathology is mostly linked with vascular dysfunction or other neurodegenerative disorders [153]. Another clinical trial found that only 28% of 63 patients with mild cognitive impairment had pure AD and about 24% had mixed dementia [154]. These studies imply the existence of significant overlaps between AD and VaD. Thus, rather than separating AD from VaD, comprehensive research should be undertaken to better understand the pathophysiology of dementia and develop therapeutic strategies.

AD patients develop an early neurovascular dysfunction, progressive neurodegeneration, selective neuronal loss, and Aβ and NFT accumulation in the brain [155,156]. Vascular damage and Aβ accumulation exert a potent synergistic effect on tau hyperphosphorylation, accelerating neuronal loss [144]. Interestingly, depending on the stage of the disease, AD affects all the cell types of the NVU, including endothelial and mural cells, glia, and neurons [156]. Besides, genetics, vascular risk factors, environmental variables, and lifestyle choices can all cause vascular diseases [156]. The neurovascular theory of AD also suggests that cerebrovascular dysfunction and the disturbance of neurovascular integrity play a role in the beginning and progression of cognitive decline and that the pathogenic events leading to AD converge at cerebral blood vessels [156]. 

### 5.6. Comparing BBB Dysfunction in AD vs. Other Neurodegenerative Diseases 

Across all neurodegenerative diseases, BBB dysfunction and disruption is a common hallmark that can be seen as a critical component of their pathogenesis (Table 2). For instance, BBB dysfunction is involved in the etiology of PD, the second most common neurodegenerative disease, which is characterized by the accumulation of α-synuclein and the loss of dopaminergic neuron loss in the substantia nigra [157]. In some patients, components of the peripheral immune system may have a role in disease development if the BBB is disturbed. Huntington’s disease (HD), an autosomal dominant neurodegenerative disease with motor, cognitive, behavioral, and metabolic disorders caused by the aggregation of the mutant Huntingtin protein, also presents BBB deficits and neurovascular abnormalities [158,159]. In addition, T-cell, B-cell, and macrophage trafficking via a faulty BBB serves pathogenic features for both amyotrophic lateral sclerosis (ALS) and multiple sclerosis (MS) [160,161,162]. 

## 6. BBB Dysfunction as a Tool for the Identification and Prediction of Early Cognitive Decline

The physiological features of the BBB strictly control the normal microenvironment necessary for appropriate neuronal activity. Any impairment either at the cellular or molecular level results in BBB breakdown and dysfunction, which causes cognitive impairments and neurodegeneration and contributes to age-related disorders. Therefore, BBB breakdown can be utilized as a predicting mechanism for various neurological disorders, such as AD and PD.

The health of the cerebral vasculature is crucial in the progression of neurodegenerative diseases. Thus, prevention interventions could focus on improving neurovascular health. Although most studies focus on removing neurotoxic proteins, early interventions maintaining BBB integrity, such as the promising work on NAD^+^ levels restoration, could help increase the healthy life expectancy. 

MRI and other imaging techniques play an essential role in healthcare decisions. The lack of MRI facilitation during the early stages of BBB disruption, on the other hand, requires the development of new approaches to BBB breakdown detection. In this context, the identification of peripheral blood markers may be one of the most effective and readily available methods for determining the BBB status. However, the blood/CSF albumin ratio cannot distinguish between BBB and blood–CSF permeability or localize leaks, according to the existing studies. Hence, approaches that can directly identify and pinpoint these illusive permeability values are needed. Acquiring a better understanding of BBB disruption across time requires conducting studies spanning wider age ranges. Including adults between the ages of 20 and 30 could provide a baseline for BBB leaking later in life. 

A recent DCE-MRI study revealed that healthy older adults had a higher BBB permeability index Ktrans than young healthy adults in the hippocampus CA1 and dentate gyrus, but not in CA3 [47]. Besides, this study showed that BBB integrity in the hippocampus deteriorated with age [47]. Another DCE-MRI study, using CSF biomarkers, found that older adults with previous cognitive impairment had higher BBB permeability than healthy people [49]. A different study used a novel MRI technique called diffusion-prepared arterial spin labeling to assess the water exchange across the BBB and detected modest BBB dysfunctions associated with reduced Aβ clearance in the CSF of healthy individuals [169]. They also reported that a decreased water exchange rate across the BBB was linked to lower cerebrospinal Aβ concentrations in various brain regions (whole brain, frontal lobe, parietal lobe, and precuneus) relevant to AD [169]. 

Furthermore, including novel ligands in positron emission tomography investigations has recently allowed the in vivo evaluation of unique BBB transport networks. For example, a study utilizing radiolabeled verapamil, a ligand for the P-glycoprotein BBB transport system, showed that healthy older adults exhibited lower activity than younger controls [170]. Besides, technological advances are required to evaluate if neurovascular dysfunction and BBB breakdown can be detected in the living brain before the onset of the entire spectrum of neurological symptoms.

## 7. Therapeutic Implications in BBB Impairment

In complex neurodegenerative disorders such as AD, the relationships between neurovascular integrity, brain structural and functional connectivity, cognitive function, and neurological symptomatology are paramount. However, they have yet to be directly explored in the most relevant in vivo context, which has only recently become possible thanks to the development of novel state-of-the-art neuroimaging and molecular biomarker approaches. Clinical research in this area will advance our knowledge and help us better grasp the link between BBB disintegration and cognitive impairment. Furthermore, a better understanding of the vascular system, promoting neurovascular health, and finding ways to maintain BBB health will also aid in the development of new, efficient cognitive impairment and neuronal loss treatments

There are numerous transporters at the BBB that could be targeted to transport drug molecules to the brain. For instance, caffeine is transported by adenosine transporter to lower the Aβ burden [171], and BBB reinforcement after caffeine supply or treatment with rivaroxaban, was shown to inhibit neurotoxic symptoms such as neuroinflammation, neurodegeneration, and object cognitive impairments in 5XFAD mice [102]. Antioxidants such as vitamin E, vitamin B, α-lipoic acid, and N-acetylcysteine are transported across the BBB by various transporters [172]

Glucocorticosteroids are currently the sole suitable and most extensively utilized therapeutic strategy to improve BBB integrity and regulate undesired inflammatory responses [173]. Another intriguing treatment method in clinical trials is based on a multipotent non-hematopoietic cell type called mesenchymal stromal cells; they have immunomodulatory, protective, and regenerative effects on injured tissue and are thus of interest for neurodegenerative diseases [174]. Inducing tight junction proteins production after BBB breakdown is another intriguing approach that could speed up BBB re-establishment [175,176]. Therapeutic BBB modulation has shown promising results against AD. FPS-ZM1, a RAGE antagonist, limits Aβ entrance into the brain, reducing neuroinflammation [177]. Recent studies have demonstrated that inhibiting the mammalian/mechanistic target of the rapamycin (mTOR) pathway with rapamycin has considerable neuroprotective effects in mouse models of AD, enhancing cerebrovascular and cognitive function. According to Van Skike et al. (2018), blocking the mTOR pathway can maintain BBB integrity via increasing the expression of tight junction proteins and decreasing the activity of matrix metalloproteinase-9, thereby restoring cognitive function [178]. 

Metal concentrations in the brain parenchyma of patients with AD are higher, resulting in free radical production and increased neuronal damage, and metal chelator nanoparticles that use the transferring receptor to cross the BBB can represent a treatment strategy for lowering the oxidative stress [179]. The success of such nanoparticles relies on their ability to diffuse through disrupted tight junctions for targeted delivery to the sites of pathology without any prerequisite of drug lipophilicity or drug transport system. Bana et al. (2014) also successfully created a nano-device to change Aβ’s morphology and cure AD [180]. They investigated how liposomes bifunctionalized with phosphatidic acid and a modified ApoE-derived peptide (mApoE-PA-LIP) affected the aggregation/disaggregation properties of Aβ. Experiments using dually radiolabeled LIP revealed that bifunctionalization improves radioactivity transit across the BBB both in vitro and in vivo (in healthy mice). Hence, liposomes have the potential to become clinically useful nanodevices for the treatment of AD. A recent study reported that a combination of etodolac and α-tocopherol improved BBB integrity and amyloid clearance, emphasizing the critical role of deficient BBB integrity in the pathophysiology of AD [181]. Furthermore, donepezil, an acetylcholine esterase inhibitor, has been recently reported to reduce injury-induced BBB disruption by elevating claudin-5 expression [182]. 

## 8. Limitations of Current Studies and Future Challenges

At present, lowering the risk of AD is primarily dependent on lifestyle modifications as well as the treatment and prevention of medical conditions. A molecule with good potency, selectivity, and bioavailability is not enough as a therapy to treat AD. To elicit the pharmacologic action required to produce the intended therapeutic benefit, it must also be able to cross the BBB and reach a particular concentration for a specific duration in the brain ISF. Compounds with the requisite physicochemical characteristics that enable transcellular transit by simple diffusion or appropriate pharmacophore that enable receptor-mediated endocytosis to enter the CNS. As most utilized drugs are P-gp substrates, an increase in P-gp substrates would promote the accumulation of several CNS-based drugs in the brain [183]. However, TNF-α enhances P-gp activity, which reduces the accumulation of P-gp substrates in the brain, resulting in restricted drug access to the brain [184]. This either increases or decreases drug access to the brain, making patients with AD more vulnerable to the development of delirium. This emphasizes how critical it is to consider the BBB in all neuropharmaceutical studies. The BBB lowers the entry of molecules that govern brain homeostasis and filters out the remainder to protect the brain, which limits the delivery efficacy of AD drugs. To date, over 400 compounds have been examined in several AD models [185]. Inadequate trial design and ignorance regarding the BBB are considered the key flaws in pharmacological trials [186].

Not inventing new drugs, but directing them to the brain by crossing the BBB is one of the most difficult challenges in AD treatment. A substantial research effort has resulted in the development of limited methods of exploitation. However, changes in BBB reveal indications in the beginning and during the progression of AD, affecting therapy and control measures. Hence, it is critical to maintain a balance between the discovery of novel compounds and the development of drug delivery technologies with greater specificity and efficacy in delivering drugs to the CNS of patients with AD.

## 9. Conclusions

Understanding which components of BBB malfunction are mending and which portions are pathogenic is an important issue to consider moving ahead. Current research should focus on determining the degree of inflammation in the brain or blood that can cause BBB dysfunction, leaks, or even destruction, and on how to protect or heal a broken BBB. Drug delivery into the CNS that can cross the BBB without disrupting function should also be considered. We anticipate that imaging techniques such as 7T MRI and other technologies will increase our ability to identify BBB abnormalities in humans and understand how they relate to blood flow changes, structural and functional brain connection changes, and cognitive and motor deficits in various populations.

## Figures and Tables

**Table 1:** Genotypic and phenotypic characteristics of transgenic mouse strains commonly used in AD research.

Transgenic Mouse Strain	Genetic Background	Disease Status	References
5XFAD	(C57BL/6 × SJL)F1 and C57BL/6 J	Amyloid plaques, accompanied by gliosis; Neuron loss occurs in multiple brain regions, beginning at approximately 6 months in the areas with the most pronounced amyloidosis; Mice display a range of cognitive and motor deficits.	[97]
APP/PS1	C57BL/6J	Amyloid plaque formation in the neocortex begins around 6 weeks of age. At 3–4 months, deposits are observed in the hippocampus, and at 4–5 months, deposits appear in the striatum, thalamus, and brainstem; All congophilic amyloid deposits have phosphorylated tau-positive neuritic processes.	[98]
Tg2576	C7BL/6;129 × 1/SvJ;129S1/Sv	Exhibit age-associated cognitive deficits such as impaired spatial learning and deficits in working memory as well as contextual fear conditioning at <6 months of age.	[99]

**Table 2:** Representative changes in BBB in neurodegenerative diseases.

Neurodegenerative Diseases	Representative BBB Changes	References
Alzheimer’s disease	Accumulation of fibrinogen, thrombin, albumin, IgG, and hemosiderin in the cortex and hippocampus; loss of pericyte capillary coverage and numbers in the cortex and hippocampus; reduced tight junction proteins; RBC extravasation and peripheral macrophage infiltration; neutophil infiltration; and increased levels of angiogenic proteins	[100,111,119,120]
Parkinson’s disease	Accumulation of fibrinogen in the striatum as well as IgG and hemosiderin in the globus pallidus; microvascular degeneration, reduced and disrupted tight junctions, altered capillary basement membrane in the subthalamic nucleus; extravasation of RBCs in the striatum; increased endothelial cell numbers in the substantia nigra	[163,164]
Huntington’s disease	Leakage of fibrin in the putamen; decreased and disrupted tight junction protein expression in the putamen; accumulation of Huntingtin protein aggregates in the endothelial cells	[158,159]
Amyotrophic lateral sclerosis	Leakage of fibrinogen, thrombin, IgG, collagen type IV, and iron-containing proteins; loss of pericytes in the medulla; microvascular degeneration, intracellular vacuolization; reduced and disrupted tight junctions; and enlarged perivascular spaces	[160,165,166,167]
Multiple sclerosis	Fibrinogen leakage; decreased and disrupted tight junctions; and leukocyte infiltration	[168]
